# Improving and sustaining the site investigator community: Recommendations from the Clinical Trials Transformation Initiative

**DOI:** 10.1016/j.conctc.2019.100462

**Published:** 2019-10-17

**Authors:** Christopher B. Fordyce, Kaitlin Malone, Annemarie Forrest, Terri Hinkley, Amy Corneli, James Topping, Matthew T. Roe

**Affiliations:** a Duke Clinical Research Institute, 2400 Pratt St, Durham, NC, 27705, USA; b Division of Cardiology, University of British Columbia, Vancouver, British Columbia, Canada; c Amgen, One Amgen Center Drive, Thousand Oaks, CA, 91320, USA; d Clinical Trials Transformation Initiative, 200 Morris St, Durham, NC, 27701, USA; e Academy of Medical-Surgical Nurses, 200 E Holly Ave., Sewell, NJ, 08080, USA; f Department of Population Health Sciences, Duke University, 215 Morris Street, Durham, NC, 27701, USA

**Keywords:** Clinical trials, Investigator turnover, Investigator retention, Research infrastructure, BMIS, Bioresearch Monitoring Information System, CRO, contract research organization, CTTI, Clinical Trials Transformation Initiative, FDA, Food and Drug Administration, PI, Principal Investigator, SOP, standard operating procedure, US, United States

## Abstract

The Clinical Trials Transformation Initiative (CTTI) Strengthening the Investigator Community Project was prompted by the need to understand the reasons for high rates of turnover among investigators who lead US Food and Administration-regulated clinical trials at research sites. Because investigator knowledge and experience directly affect the quality and ultimate success of clinical trials, investigator turnover has important implications for the research enterprise, as well as the patients and other stakeholders who depend on the outcomes of clinical research. The CTTI project team used findings from both quantitative and qualitative research activities, as well as input from an expert meeting with multiple stakeholders, to delineate key concerns faced by investigators and recommend practical, action-based solutions. The recommendations focus on strengthening four key categories of site-based research activity: developing site-based research infrastructure and staff, optimizing trial execution and conduct, improving site budget development and contract negotiations, and discovering opportunities for conducting additional trials.

## 1 Introduction

High attrition rates for investigators working on United States (US) Food and Drug Administration (FDA) regulated trials—and the resulting need to continuously recruit and train new investigators—increase the costs of performing clinical trials and threaten the quality and efficiency of trial conduct [1]. Analyses of Form FDA 1572s (“Statement of Investigator”) suggest that turnover among investigators working on FDA-regulated trials is increasing [2]. One recent examination of data contained in the US FDA's Bioresearch Monitoring Information System (BMIS) database found that from 1999 to 2015, the number of clinical trial investigators submitting a Form FDA 1572 declined by approximately one-third [2]. Furthermore, evidence suggests that many investigators are leaving clinical research due to difficulty balancing workload, time requirements, data and safety reporting burdens, and financial issues [3].

Knowledgeable and experienced site investigators are vital to conducting efficient, high-quality clinical trials, and substantial time and resources are needed to initiate and train new site investigators. To date, little is known about why investigators stop conducting site-based research or strategies necessary to overcome challenges frequently encountered by investigators. Our previous research found that nearly half (44%) of investigators expressed an interest in continuing to participate in clinical trials, but indicated that they lacked opportunities to do so [3]. This trend of investigator turnover and researchers being inadvertently driven away from clinical research has the potential to threaten the overall quality and efficiency of clinical trials. Because investigator knowledge and experience directly affect the quality and ultimate success of clinical trials, the answers to these questions have important implications for clinical research, patients, and other stakeholders.

To explore the reasons for this high rate of turnover, the Clinical Trials Transformation Initiative (CTTI)—a public-private partnership to develop and drive adoption of practices to increase the quality and efficiency of clinical trials—developed the Strengthening the Investigator Site Community Project. The Investigator Community Project team gathered data through a review of the BMIS database [2], a survey of investigators who completed only one clinical trial (“one and done” site investigators) [3], qualitative interviews with experienced investigators who have completed multiple prior clinical trials, and a multi-stakeholder meeting convened by CTTI.

In this manuscript, we summarize the findings from these evidence-gathering activities that informed our work and present a series of practical, action-based recommendations and resources to facilitate the engagement, training, and retention of qualified site investigators and research personnel in four domains: 1) developing site-based research infrastructure and staff, 2) optimizing trial execution and conduct, 3) improving site budget and contract negotiations, and 4) discovering opportunities for conducting additional trials.

## 2 Evidence gathering

### 2.1 BMIS database review

An observational database study was conducted using information downloaded from BMIS on November 14, 2016. This study examined overall trends in the clinical investigator workforce and trends within specific “phenotypes” based on level of engagement. The study also explored differences associated with investigator location (U.S.-based or non-U.S.-based). Full details of the methods and results of this survey have been described previously [2]. Briefly, investigators were stratified into 1 of 3 “phenotypes:” “one and done” – investigators with 1 Form FDA 1572 submission across the study interval; “stop and go” – investigators with at least 2 submissions for whom the interval between first and second submission occurred beyond the 75th percentile; and “stayer” – investigators with at least 2 submissions for whom the interval between first and second submission occurred within the 75th percentile.

Of 172,453 unique investigators who submitted a Form FDA 1572 from 1999 to 2015, 49.6% were classified as one-and-done investigators; 12.6% as stop-and-go investigators; and 38.7% as stayer investigators. Over the study duration, the total number of investigators conducting FDA-regulated drug trials declined by approximately one third. The largest absolute and proportional declines of all subgroups occurred for stayer investigators; the number of stop-and-go investigators also declined. In contrast, the number of one-and-done investigators grew across the study period. CTTI recognized that these findings may signal adverse trends in the clinical investigator workforce.

### 2.2 Survey

**Fig. 1:**
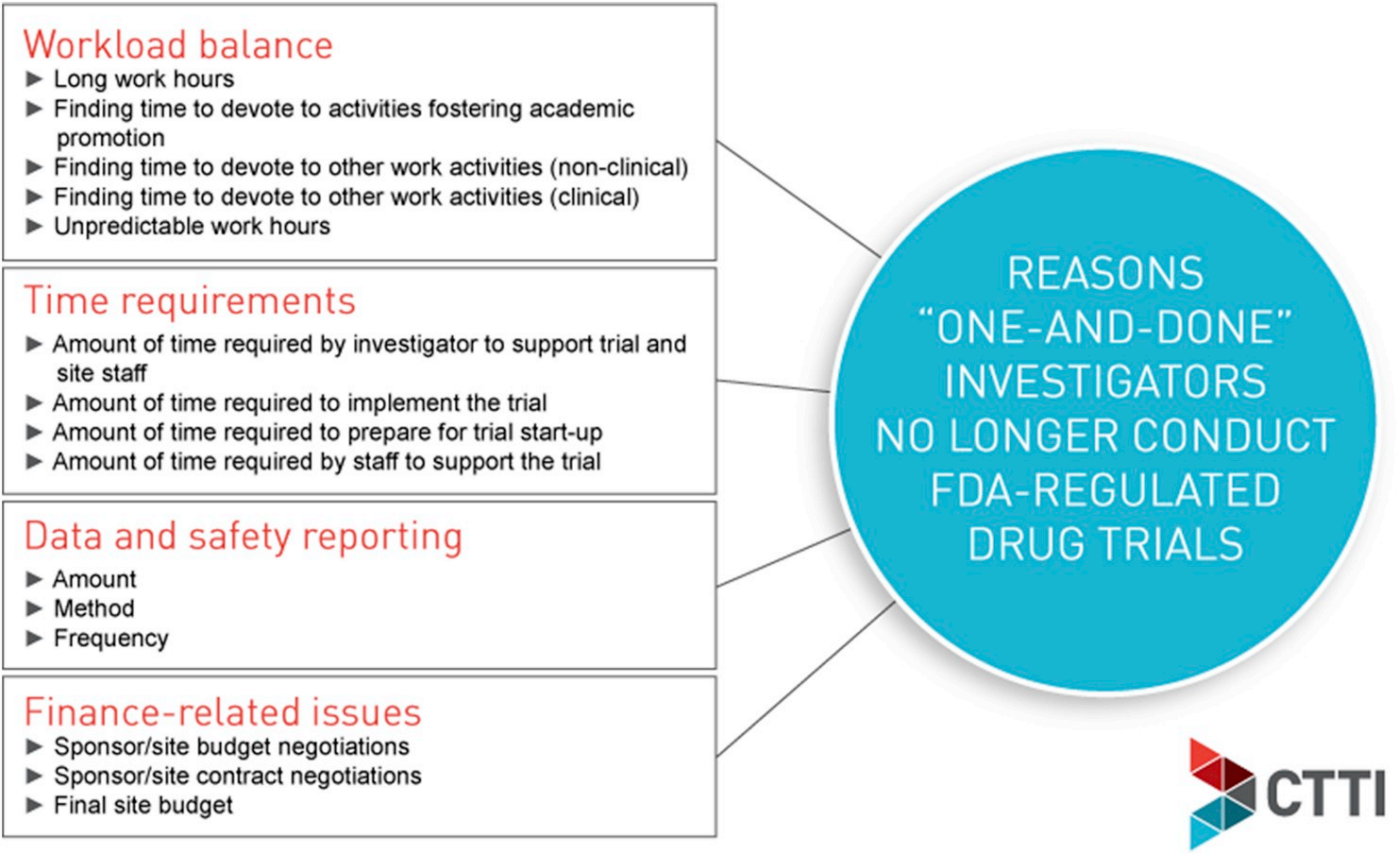
Survey results: Reasons “one and done” investigators No longer conduct FDA-Regulated drug trials.

Subsequently, a survey was administered to US-based Principal Investigators (PIs) between October 7 and November 5, 2015. The survey sought to identify challenges and reasons why one-and-done investigators did not pursue a PI role in subsequent clinical trials. The methods and results of this survey are have been described in detail by Corneli et al. [3]. In brief, 28.9% of one-and-done investigators cited personal reasons for not conducting another trial, while 44.4% wanted to conduct another trial but did not have the opportunity to do so. The reasons provided varied significantly between academic and non-academic/community-based investigators. Academic investigators were more likely to indicate they were no longer participating due to of a lack of available trials, whereas community investigators were more likely to cite personal choice as the reason for not participating. In addition, the survey identified three broad categories of barriers to continued participation in FDA-regulated trials: 1) difficulty with workload balance, 2) time requirements for conducting trials, and 3) burdens imposed by data and safety reporting requirements. Dissatisfaction with trial finance also influenced decisions for many investigators (Fig. 1).

### 2.3 Interviews

Qualitative, in-depth interviews were conducted from July 20, 2016 to October 12, 2016 with a group of purposefully selected site investigators who had completed multiple FDA-regulated clinical trials and remained active in conducting such trials. Detailed methods and findings will be described elsewhere. In brief, among other findings, active investigators reported that they were successful at participating in multiple FDA-regulated drug trials because they had 1) sufficient and well-trained staff, particularly clinical research coordinators, 2) strong commitment and work ethic, 3) institutional support, 4) the ability to recruit patients, 5) business knowledge and experience, 6) a strong reputation, 7) the ability to network, and 8) the ability to be realistic when selecting protocols.

### 2.4 Expert meeting

On April 5, 2017, the Investigator Community Project held a one-day stakeholder meeting in Silver Spring, Maryland to discuss and draw conclusions from the survey and interview data. The meeting convened over 25 participants, representing patient advocates, academic medical centers, private practice, industry (including pharmaceutical, medical device, and contract research organizations), and government (including the National Institutes of Health and the US FDA). The CTTI project team presented project findings, and participants then discussed evidence-based solutions to inform development of project recommendations and resources.

At this stakeholder meeting, experts discussed the survey and interview results and identified 5 key themes: 1) although clinical site investigators and their study staff are often interested in continuing to participate in clinical trials, they need access to training and supportive infrastructure; 2) smaller clinical practices sometimes struggle to establish themselves with research sponsors and CROs and to continue to attract sufficient interest from these organizations when new trials are launched; 3) education and supportive resources are particularly required for smaller practices and researchers outside of academic institutions; 4) increased communication, education, and transparency could improve site performance; and 5) incorporating input from all stakeholders can improve the process of protocol development, thereby enhancing the overall trial experience and reducing delays in study startup with better protocols that are easier to implement [4].

### 2.5 Development of recommendations and resources

After the project team determined that the evidence-gathering objectives were met and that no major gaps in data remained, they followed an iterative development process to craft a series of project recommendations and resources for all clinical research stakeholders to strengthen the site investigator community and the clinical research ecosystem.

First, team members used the data from the evidence-gathering activities and input from the expert meeting to draft and propose project recommendations and resources. The project team then incorporated input from the CTTI Executive Committee Champion, CTTI leadership, CTTI members, and an ad hoc committee comprised of change agents independent of the project team. Refinement of the recommendations and resources continued until the team reached consensus. Prior to dissemination, the CTTI member organizations [5] reviewed all project recommendations and resources and provided constructive feedback and edits. Finally, the CTTI Executive Committee approved the recommendations and resources. The methods employed for this process have been described in further detail in a previous paper [6].

## 3 Recommendations and resources

**Fig. 2:**
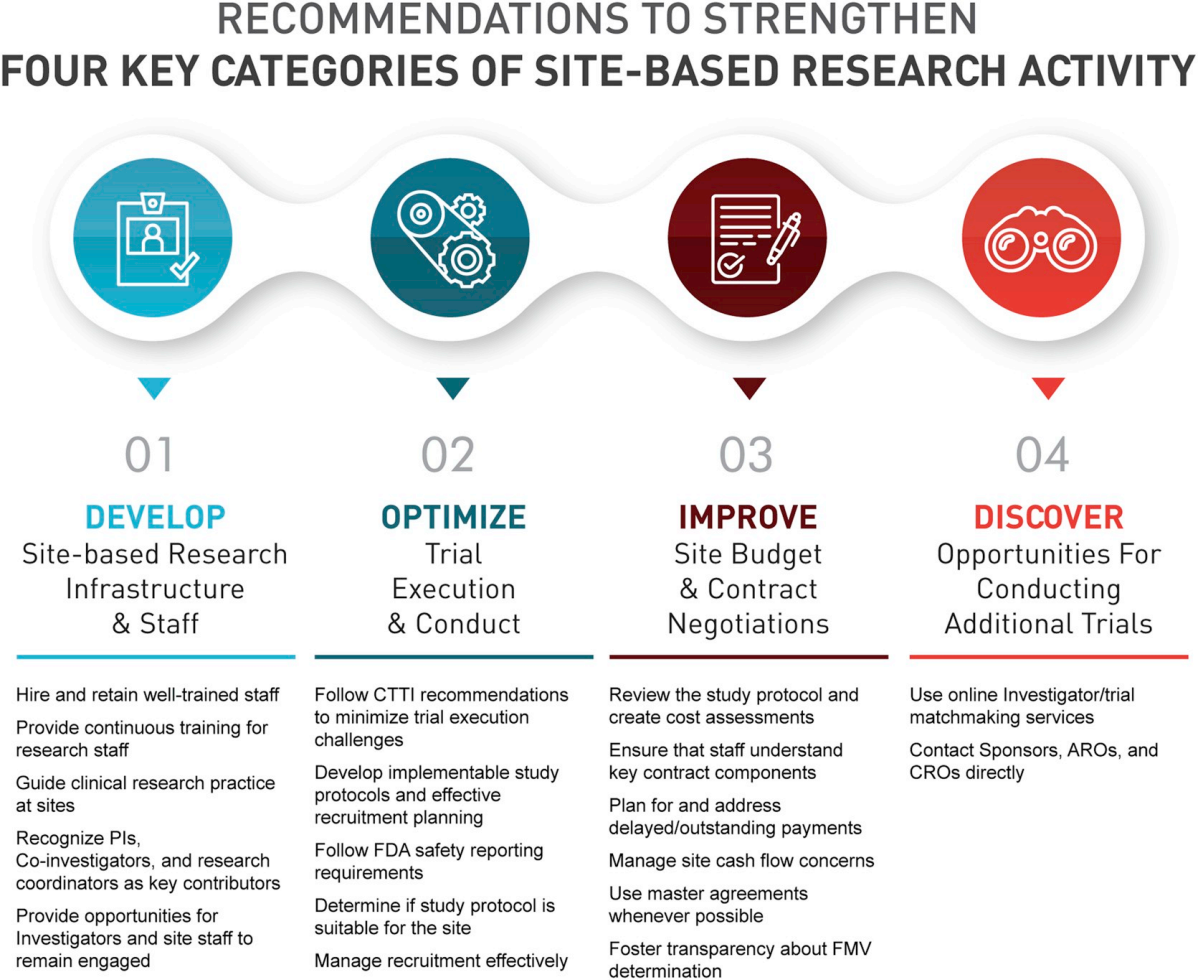
CTTI recommendations snapshot.

Using the iterative process described above, the Investigator Community Team made the following recommendations, which focus on strengthening four key categories of site-based research activity: 1) developing site-based research infrastructure and staff, 2) optimizing trial execution and conduct, 3) improving site budget development and contract negotiations, and 4) discovering additional trials to conduct (Fig. 2).

### 3.1 Developing site-based research infrastructure and staff

#### 3.1.1 Recommendations for investigative sites

Site investigators must have foundational knowledge to carry out their roles [7] but also require supportive infrastructure and well-trained site research staff to conduct high-quality clinical trials while effectively managing the workload and other burdens associated with site-based research. Regardless of whether investigators are embedded within large academic or private health systems, or work in smaller community/private-practice settings, institutional support for their research activities is critical to their success.

• **Hire and retain well-trained staff.** At investigative sites, it is important to hire and retain well-trained, experienced research coordinators and other essential research staff. Research coordinators perform many essential trial-related activities and are thus essential for site investigators to be successful in trial recruitment and conduct.
• **Provide continuous training for research staff.** Research sites must value and support training for site research personnel. Targeted training specific to site-based clinical research must include all staff involved in clinical research activities, and not solely the investigator. Access to and participation in training and educational opportunities can be facilitated through online training courses, onsite mentoring from organizations that conduct clinical trials, including CROs,^1^ and participation in professional organizations that support site research professionals.
• **Guide clinical research practice at sites with standard operating procedures (SOPs) and systems.** Systems (prompts and data entry within electronic health records; site-focused clinical trials management systems, etc.) and written SOPs for supporting site-based research can help ensure compliance and consistent, high-quality execution of clinical trials. Sites may find it helpful to organize study-related procedures and tasks using these systems, according to key trial milestones: pre-study, study start-up, study execution, and study close-out.

#### 3.1.2 Recommendations for sponsors, CROs, and health systems/private practices

• **Recognize PIs, co-investigators, and research coordinators as key contributors to product development.** Sponsors, CROs, and health systems/private practices, as well as their delegates, should formally acknowledge site investigators and site research staff who conduct clinical research. This may include approaches such as thanking site staff personally or recognizing contributions of site staff and study participants via websites, or in dissemination of trial results, publications, and presentations. Also, site investigators should be given more opportunities to participate as co-authors on publications resulting from completed clinical trials, presuming that they fulfill standard International Committee of Medical Journal Editors authorship requirements.
• **Provide opportunities for investigators and site staff to remain engaged between trials.** Inactivity between active trials presents challenges for maintaining and supporting site-based research staff but also opportunities for sustained engagement with organizations that support and conduct trials. Sponsors, CROs, and health systems/private practices should actively support investigators and research site staff between trials by providing developmental opportunities via attendance at clinical trial-related conferences, continual medical education-certified trainings, and engagement with professional society and trade associations.

### 3.2 Optimizing trial execution and conduct

#### 3.2.1 Recommendations for sponsors

Investigators and site staff should include operational considerations as part of overall preparations for conducting a successful trial. Sponsors should follow CTTI Quality by Design [8] and Recruitment Project [9] recommendations to minimize trial execution challenges.

• **Develop implementable study protocols and ensure effective recruitment planning.** Attention to minimizing recruitment challenges at the trial design and protocol development stages is essential to improve clinical trials conduct. This goal can be achieved by engaging all trial stakeholders as equal partners in the protocol development process, ensuring the relevance of the scientific question to all stakeholders, limiting protocol complexity to reduce the burden of participation, developing realistic trial eligibility criteria, and limiting the burden of data collection for trial-specific data to include only those data needed to maintain patient safety and address the scientific questions contained within [10,11].
• **Follow FDA safety reporting requirements.** The FDA's requirements for reporting safety issues and adverse events impose critically important obligations, as well as burdens, on site investigators. Creating, reviewing, and dispatching adverse event reports can require significant time and effort, despite FDA efforts to minimize sponsor and site burdens in this area [12,13]. Following referenced federal guidance and CTTI recommendations will help to foster a reduction in the associated regulatory safety reporting workload.

#### 3.2.2 Recommendations for investigative sites and health systems/private practices

• **Determine whether the study protocol is suitable for the site.** Investigators, site staff, and associated health systems should review and assess the study protocol for basic feasibility [13] and make informed decisions about participating in trials based upon realistic expectations regarding trial implementation and recruitment. Above all, investigators and health systems/private practices should be selective in participating in trials and decline trials that do not appear to be feasible for their patient population and resources (e.g., staff capacity and competing trials).
• **Manage recruitment effectively.** To ensure successful, sustainable trial recruitment, investigators and staff should seek out potential patient perspectives on trial participation, communicate with trial sponsors about any concerns related to recruitment (e.g., eligibility criteria and burden of the trial's schedule of assessments), and discuss any challenges in screening or recruiting participants with site staff to identify strategies to overcome them. It is also recommended that sites develop a viable recruitment plan prior to study execution, recruit patients from their own clinical practices, and develop an effective patient referral system (e.g., local physicians, community centers, religious centers, and health centers) to improve trial recruitment. Finally, it is important to experiment with different recruitment strategies, track results, and develop new approaches to boost recruitment rates.

### 3.3 Improving site budget and contract negotiations

Issues related to contracts and budgets present challenges for site investigators and sponsors. Stakeholders often have differing perspectives regarding the adequacy and accuracy of site budget allocations, the fairness of budget and contract negotiations, the optimal schedule of payments, and the methods by which fair market value is determined and applied to budget estimates and projections for individual sites.

#### 3.3.1 Recommendations for investigative sites

Investigators and site personnel should focus on four critical areas related to negotiating and executing budgets and contracts for trials, addressing payment delays, and managing cash flow:

• **Review the study protocol and create cost assessments.** When negotiating the trial budget, site research staff should review the study protocol/schedule of assessments, create their own cost assessments, and supply justifications for fair costs based upon their realistic estimates of time and resource allocation to execute the trial.
• **Ensure that staff understand key contract components.** Site research staff should all understand key components of contract terms and/or ensure that qualified financial experts develop their budget estimates and negotiate their local site budgets.
• **Plan for and address delayed/outstanding payments.** Investigators should develop specific strategies to address delayed and/or outstanding payments.
• **Manage site cash flow concerns.** Investigators should identify and incorporate strategies to manage site cash flow concerns.

#### 3.3.2 Recommendations for sponsors and CROs

• **Use master agreements whenever possible.** Master agreements can greatly expedite the process of contracting across multiple trials and provide clarity to sponsors, CROs, and investigative sites. Sponsors should consider more frequent use of master agreements with sites and should create a template set of key administrative elements for site contracts and associated research terms to facilitate the execution of master agreements.
• **Foster transparency about fair market value determination.** Sponsors and CROs should provide sites with a transparent accounting of how fair market value is determined. Fair market value calculations (benchmarked averaged estimates of procedure costs and associated reimbursement) often are not fully understood by sites and can become a source of mistrust. More transparent articulation from sponsors and CROs on fair market value calculations may foster improved dialog and smoother contract negotiations between sponsors, CROs, and investigative sites.

### 3.4 Discovering opportunities for conducting additional trials

A large proportion of site investigators indicate that they want to conduct additional trials but do not know how to access opportunities for doing so [10]. Interested investigators should consider using the multiple professional societies, trade associations, and companies that provide investigator/trial matchmaking services. These online systems match qualified investigators and sites with sponsors and CROs around the world.

Investigators who want to pursue opportunities for conducting additional trials should also contact sponsors and CROs directly. Many sponsors and CROs have online registration portals for investigators interested in conducting their clinical trials. Investigators should also consider completing online profiles on these portals for sponsors and CROs conducting studies in their therapeutic area of expertise. Additionally, organizations that represent clinical trial stakeholders should collaborate to freely source information about trials so that all interested and qualified sites (and their patients) can have access to important trials that are evaluating therapies that address unmet clinical needs and conditions that need better treatment options.

## 4 Conclusions

Challenges faced by site investigators that have the potential to threaten quality and efficiency of FDA-regulated clinical trials persist. Through discussion with multiple stakeholders, including trial sponsors, CROs, and clinicians, and the use of both quantitative and qualitative research approaches, we have delineated key concerns faced by investigators, and recommended several stakeholder-specific solutions to improve the investigator experience and clinical trial conduct. Recommendations include streamlining research infrastructure and staff, clarifying site budget and contract negotiations, and providing more trial opportunities for investigators. This multi-faceted approach should be considered to improve the conduct of clinical trials.

## Funding

Funding for this manuscript was made possible, in part, by the US Food and Drug Administration through grant R18FD005292 and cooperative agreement U19FD003800. Views expressed by the authors do not necessarily reflect the official policies of the Department of Health and Human Services, nor does any mention of trade names, commercial practices, or organization imply endorsement by the US Government. Partial funding was also provided by pooled membership fees and in-kind contributions from CTTI's member organizations.

## Footnotes

1 We use the term contract research organization, or CRO, throughout this paper as a term to encompass both CROs and academic research organizations.
